# Effectiveness of Atomoxetine and Stimulant Combination in Attention-Deficit/Hyperactivity Disorder (ADHD) Treatment: A Systematic Review

**DOI:** 10.7759/cureus.79378

**Published:** 2025-02-20

**Authors:** Tom Cheng, Andrew J Boileau

**Affiliations:** 1 Neuroscience and Neurology, Saba University School of Medicine, The Bottom, BES

**Keywords:** adherence to medication, adjunct therapy, atomoxetine, attention deficit hyperactivity disorder (adhd), augmentation therapy, cgi-s, combination drug therapy, methylphenidate (mph), non-stimulant

## Abstract

Attention-deficit/hyperactivity disorder (ADHD) is a neurodevelopmental disorder that can present with inattention, impulsivity, hyperactivity, or any combination of the three. For ADHD treatment, stimulants are widely considered first-line medications, with methylphenidate formulations being the most commonly prescribed. In patients who do not tolerate stimulants at higher doses, non-stimulant augmentation is a potential treatment strategy. Atomoxetine, as the first approved non-stimulant for ADHD by the United States Food and Drug Administration, has also been evaluated in several studies for combination therapies over the years. This review aims to investigate the effectiveness of combining stimulants such as methylphenidate with atomoxetine based on all newer studies, which came after the last major systematic review published in 2013. Six retrospective studies were included based on eligibility criteria among all studies published from 2012 to 2024 that were relevant to the research question. Three out of the six studies contained data regarding efficacy: one reported no significant difference in ADHD severity reduction between atomoxetine monotherapy and combination therapy overall, while two reported significantly decreased ADHD severity among treatment-resistant monotherapy patients after switching to combined methylphenidate and atomoxetine therapy. Four out of the six studies measured adherence: three reported significantly greater adherence with combined therapy versus monotherapy, while one reported that concomitant prescription of atomoxetine with methylphenidate significantly contributed to methylphenidate discontinuation. Limitations to this review include a limited quantity of total studies relevant based on the defined research question and the eligibility criteria, and lower certainty of evidence among them due to study limitations and even conflicts of interests. The heterogeneity in definitions and metrics carries over into population characteristics and analyses of each study, all of which contribute to the overall indirectness of evidence toward the defined question. For future studies, the current research question should be narrowed down to investigate specific ADHD patient subpopulations, with standardized protocols with consistent definitions and statistical methods to conduct prospective randomized controlled studies across multiple sites with longer follow-up periods, to have more meaningful answers regarding optimal combinations of atomoxetine with various stimulant formulations for long-term outcomes.

## Introduction and background

Attention-deficit/hyperactivity disorder (ADHD) is defined in the Diagnostic and Statistical Manual of Mental Disorders, Fifth Edition (DSM-5) as “a persistent pattern of inattention and/or hyperactivity-impulsivity that interferes with functioning or development” [1]. As of 2020, ADHD is estimated to have a worldwide prevalence of 5.29% among children and adolescents and 2.5% among adults between 19 and 45 years [2]. Although ADHD is predominantly seen as one of the most common conditions affecting childhood learning and development, many cases continue onto adulthood with associations of increased risk of comorbid mood disorders, anxiety disorders, substance abuse disorders, and even higher rates of incarceration. Combining the direct and indirect economic impacts associated with ADHD in the US alone yielded an estimated cost of between $143 billion and $266 billion [3].

Based on several clinical recommendations and guidelines, stimulants are considered the first-line treatment option, particularly norepinephrine-dopamine reuptake inhibitors (NDRIs). NDRIs have a long history of proven effectiveness with quick onset of effects for most ADHD patients [2,4-6]. Two main drugs that can be classified as NDRIs and are in use currently are amphetamine (AMP) and methylphenidate (MPH), each of which has been additionally formulated into drugs with different timed-release profiles. Amphetamine and methylphenidate formulations both primarily impede the dopamine transporters (DAT) and norepinephrine transporters (NET) at the presynaptic neuron, which reduces the reuptake of both neurotransmitters and prolongs their synaptic activity. Meanwhile, AMP also stimulates cytoplasmic dopamine release at vesicular monoamine transporters (VMAT2) [2,6]. Their shared pharmacological activity means they also share potential side effects, most commonly dry mouth, reduced appetite, and insomnia. NDRIs have also been reported to exacerbate Tourette syndrome and other tic disorders [4,6]. Despite similarities between AMP and MPH formulations, the side effects of AMPs tend to be more severe [2,6].

Among non-stimulant alternatives in ADHD medications, atomoxetine (ATX) was released in 2002 as Strattera by Eli Lilly, becoming the first non-stimulant to be approved for ADHD monotherapy by the Food and Drug Administration (FDA) in the United States (US) [7]. ATX’s primary mechanism of action is to act as a selective norepinephrine reuptake inhibitor specific to the prefrontal cortex, where it also indirectly increases dopamine residual concentrations [4]. Since the dopaminergic effects are only in the prefrontal cortex but not in the nucleus accumbens or striatum [7], there are theoretical benefits to attention and emotional regulation in the prefrontal cortex without impact on the mesolimbic reward pathway. Hence, the risk for addiction and dependence is reduced in ATX compared to stimulants, as it can be stopped abruptly without withdrawal effects, making them a preferred option for ADHD patients with a higher risk for substance use disorders. The less activating properties of non-stimulants like ATX also benefit those with comorbidities in anxiety, sleep, or tic disorders, which may all be exacerbated by stimulant use [7]. Yet, ATX’s history of an FDA black box warning for increased risk of suicidal ideation in children or adolescents should also be considered, particularly with initial dosing or with dose adjustments [4].

Rationale

The consensus among previous literature of reviews and guidelines [4-7] suggests that while stimulants remain the first line for ADHD pharmacotherapy, there are drawbacks in terms of tolerability for patients with comorbidities. Meanwhile, ATX offers the advantages of lower abuse potential and better tolerability in cases of comorbid tic or anxiety disorders, and it lacks the relatively rapid onset of effects seen with stimulants, which occurs within days rather than weeks. To balance the efficacy and tolerability of ADHD pharmacotherapy, the possibility of augmenting stimulants, such as MPH formulations, with non-stimulants, such as ATX, is a point of continued interest. The last major systematic review specific to the topic of interest was by Treuer et al. (2013) [8], which “suggests, but does not confirm, that this drug combination may benefit some, but not all, patients who have tried several ADHD medications without success.” Revisiting this topic by searching for primary studies available since the last systematic review [8] may provide further insights regarding the ADHD patient population that would benefit most from this combination of therapies.

## Review

Methods

Review and Quality Assessment Approach

The Preferred Reporting Items for Systematic Reviews and Meta-Analyses (PRISMA) 2020 guidelines were consulted in the compilation and overall structure of this review [9]. The 2019 Cochrane Handbook for Systematic Reviews of Interventions was consulted for quality assessment guidelines and best practices of any included studies regarding the risk of biases [10] and certainty of evidence [11].

The Grading of Recommendations Assessment, Development, and Evaluation (GRADE) approach was used to determine the quality of evidence by levels of certainty within each included study. Per Cochrane guidelines, all randomized trials were initially classified as “High” certainty by design, unless shown otherwise by tools such as the Cochrane Risk-of-Bias version 2 (RoB2). Likewise, non-randomized studies of interventions (NRSIs) were initially classified as “Low” certainty, due to inherent biases from lack of randomization (e.g., confounding and selection bias), unless shown otherwise by the Risk Of Bias In Non-randomized Studies of Interventions (ROBINS-I) [10,11].

Determination of eligibility criteria, search strategies, screening, and data extraction decisions was made by consensus of all authors, including resolution of any potential discrepancies in quality assessment results post-independent evaluations, with the consultation of the institutional medical librarian as an independent reviewer for arbitration if necessary.

Eligibility Criteria & Search Strategy Formulation

Systematic searches for primary sources (i.e., randomized trials and observational studies) were conducted in the databases of PubMed, Cochrane Central Register of Controlled Trials (CENTRAL), and EBSCO by search strategies with minor syntax variations. As the systematic review by Treuer et al. (2013) [8] had already covered studies up to the year 2012, only studies published between 2012 and 2024 were identified for the current review. Excel spreadsheets (Microsoft Corporation, Redmond, WA) with search results of titles in rows from each database were sorted alphabetically and manually compared with one another to identify any duplicates for removal before title/abstract screening. Exclusion criteria were established for manual title/abstract screening to filter out irrelevant studies (e.g., non-human studies, qualitative studies, studies without ADHD focus, studies without ATX + stimulant combination, etc.) prior to full-text eligibility assessment.

The first search algorithm was constructed in PubMed with the following strategy in mind: (A) the “AND” operator to link the Medical Subject Heading (MeSH) terms of “Attention Deficit Disorder with Hyperactivity” with "Drug Therapy, Combination” as broad categories. (B) The “AND” operator to link relevant generic medication names (e.g., amphetamine, lisdexamfetamine, methylphenidate, dexmethylphenidate, atomoxetine, etc.) together with synonyms related to combination therapy (e.g., concurrent, concomitant, augment, supplement, complement, etc.) all connected with the “OR” operator. (C) The “NOT” operator to exclude non-primary sources such as reviews, systematic reviews, meta-analyses, comments, and letters.

As CENTRAL was a repository for only clinical trials, the PubMed search algorithm was adjusted for syntax compatibility for Cochrane Library (i.e., "MeSH" to "mh") with the same inclusion criteria logic, without requiring the “NOT” conditions for publication types. As EBSCO did not recognize the PubMed "pt" operator for publication types, results were manually screened for publication types without the “NOT” conditions as well. The search strategies and manual filters used for each database are captured in Table 1.

**Table 1:** Search strategies and manual filters used for study identification of each database. MeSH: Medical Subject Heading; CENTRAL: Cochrane Central Register of Controlled Trials.

Database	Search strategy used	Manual filters used
PubMed	((("Attention Deficit Disorder with Hyperactivity"[MeSH]) AND "Drug Therapy, Combination"[MeSH]) OR (atomoxetine AND (stimulant* OR stimulant* OR amphetamine* OR lisdexamfetamine* OR methylphenidate* OR dexmethylphenidate*) AND (combin* OR concomitan* OR supplemen* OR augmen* OR concurren* OR complemen* OR adjunc*))) NOT (review [pt] OR systematic review [pt] OR meta-analysis [pt] OR comment [pt] OR letter [pt])	Results by year: from 2012 to 2024
Cochrane Central Register of Controlled Trials (CENTRAL)	([mh "Attention Deficit Disorder with Hyperactivity"] AND [mh "Drug Therapy, Combination"]) OR (atomoxetine AND (stimulant* OR stimulant* OR amphetamine* OR lisdexamfetamine* OR methylphenidate* OR dexmethylphenidate*) AND (combin* OR concomitan* OR supplemen* OR augmen* OR concurren* OR complemen* OR adjunc*))	As per the Advanced Search Default Settings of CENTRAL, except with search limits: CENTRAL trials only, original publication year “between 2012 and 2024” and “search word variations”
EBSCO	((("Attention Deficit Disorder with Hyperactivity"[MeSH]) AND "Drug Therapy, Combination"[MeSH]) OR (atomoxetine AND (stimulant* OR stimulant* OR amphetamine* OR lisdexamfetamine* OR methylphenidate* OR dexmethylphenidate*) AND (combin* OR concomitan* OR supplemen* OR augmen* OR concurren* OR complemen* OR adjunc*)))	As per EBSCO advanced search default settings, except publication date with custom range “01/01/2012 -> 12/03/2024”

Results

Screening Process & Included Studies

**Figure 1:**
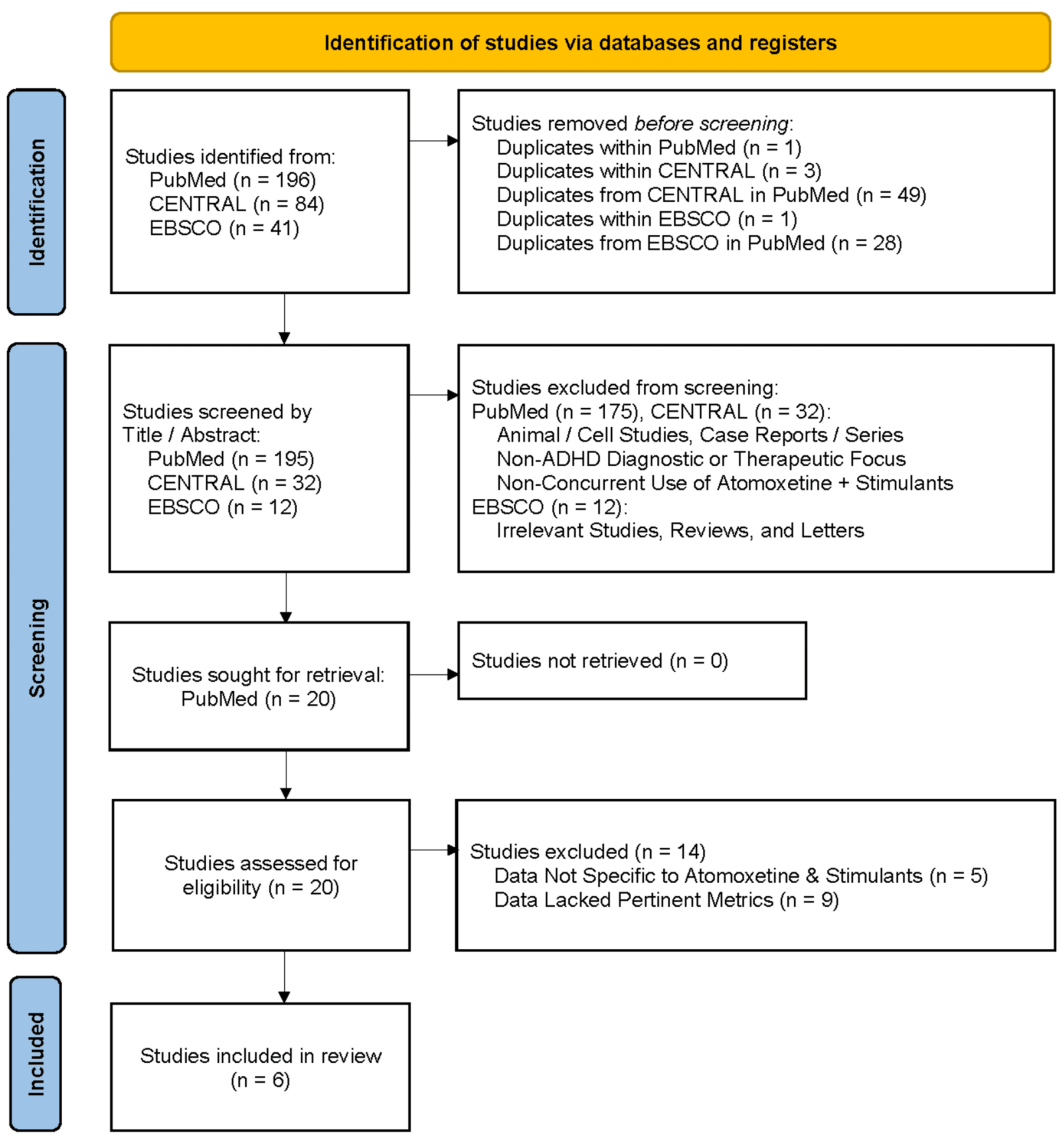
PRISMA flowchart of selected studies. PRISMA: Preferred Reporting Items for Systematic Reviews and Meta-Analyses; CENTRAL: Cochrane Central Register of Controlled Trials; ADHD: attention-deficit/hyperactivity disorder.

Out of the 321 total initial search results across the three databases, 82 total duplicate studies were removed. From the 239 non-duplicate results, 219 results were then excluded at the title and abstract screening stage, due to one or more of the following reasons: results with in vitro or animal models, results that were categorized as reviews, comments, or letters, results with diagnoses and treatment of conditions other than ADHD, or results without concurrent use of stimulants and ATX in methodology. Out of the remaining 20 results, five were excluded due to study data not being sufficiently specific to the methylphenidate and atomoxetine therapy combination, and nine were excluded due to study data lacking pertinent details for meaningful analysis. Thus, at the completion of the screening process (Figure 1), six primary articles were ultimately included in this review.

Definition of Effectiveness

Based on the heterogeneity of data available across the six included studies, it was deemed necessary for this review to establish unifying definitions and metrics where possible. Thus, this review has defined “effectiveness” as the combination of two factors, i.e., efficacy and adherence, with further internal definition below.

Definition of Efficacy

Efficacy could be most strictly defined as the ability to produce the expected desired result based on treatment within an experimental setting, such as a randomized control trial (RCT) [12]. However, the term would sometimes be applied broadly to the real-world outpatient setting, as the authors in some retrospective observational studies included in this review had done so. For simplification, the changes in ADHD signs and symptoms via standardized ratings would be referred to as “efficacy” in the context of this review. Among standardized psychometric rating systems, the Clinical Global Impressions (CGI) scale was among the most commonly used, with its ease of adaptation to various clinical settings. The Clinical Global Impressions-Severity (CGI-S) rating is based on an integer scale from 1 to 7, with the integer corresponding with the following statements: 1 = not ill; 2 = borderline ill; 3 = slightly ill; 4 = moderately ill; 5 = markedly ill; 6 = very much ill; 7 = severely ill [13]. As a decrease in CGI-S score over time reflects a decrease in ADHD severity, it could be considered a direct measure of “effectiveness” for an ADHD treatment regimen.

Definition of Adherence

Adherence could be defined as how accurately patients follow a prescribed treatment regimen over the course of an intended treatment duration. While there might be many different criteria and metrics (e.g., medication possession ratio (MPR), discontinuation vs. maintenance, etc.) to determine who is considered an “adherent” patient, the most common factor came down to duration (e.g., “length of therapy,” “duration on treatment regimen,” and “treatment duration”) in which the patient acceptably followed the overall treatment plan. As patients tended to only stay with medications with perceived therapeutic benefits, it could be considered an indirect measure of “effectiveness” for an ADHD treatment regimen.

Study Characteristics and Summaries of Findings

Among the six studies included in this review, three contained data relevant to efficacy, while four contained data relevant to adherence. An overview of all study designs and population characteristics is provided in Table 2, ordered alphabetically by the first author’s last name. The summaries of key outcomes and quality of evidence are provided in Table 3 for efficacy, and in Table 4 for adherence. Across all six studies, the common P-value of statistical significance was set at less than 0.05, while the confidence intervals (CI) were set at 95% where applicable.

**Table 2:** Overview of the study and population characteristics. ADHD: attention-deficit/hyperactivity disorder; ATX: atomoxetine; MPH: methylphenidate; US: United States; OROS-MPH: osmotic-release oral system methylphenidate.

First author (year)	Study design	Study population	Study period	Age range	Sample size	Effectiveness data extracted	Industrial funding/declared conflicts of interests
Bahn (2021) [14]	Retrospective observational (chart review)	Attention-deficit/hyperactivity disorder (ADHD) patients from one hospital in Korea were treated with either atomoxetine (ATX), methylphenidate (MPH), or both together over two or more visits.	January 2009 to December 2019	2-74	929	Adherence	None/none
Clemow (2015) [15]	Retrospective observational (chart review)	ADHD patients were prescribed ATX for the first time and for at least 50 days at the two clinical sites in the United States (US), without any ADHD-treatment naivety restrictions or washout periods.	January 2011 to December 2013	6-62	191	Efficacy and adherence	Funding by Eli Lilly/all authors are “employees and minor shareholders of Eli Lilly and Company and/or one of its subsidiaries.”
Clemow (2016) [16]	Retrospective observational (prescription data)	Adult ADHD patients with at least one pharmacy claim for ATX in an insurance database of the US, with a six-month ATX-free preindex period before prescription at index, with a one-year follow-up.	January 2006 to September 2013	18+	37,624	Adherence	Funding by Eli Lilly/five authors are “employees of Eli Lilly and Company” and/or one of its subsidiaries, while the other six authors are “members of a Lilly medical advisory board.”
Ishizuya (2021) [17]	Retrospective observational (prescription data)	Pediatric ADHD patients in the Japanese Medical Insurance Database with a one-year MPH-free period prior to first osmotic-release oral system methylphenidate (OROS-MPH) prescription and a one-year follow-up period minimum.	December 2007 to May 2015	6-17	1,353	Adherence	None/corresponding author “has received speaker’s honoraria” from Janssen, among various pharmaceutical companies.
Ozbaran (2015) [18]	Retrospective observational (chart review)	Pediatric ADHD patients from one hospital in Turkey, who switched from either ATX or MPH monotherapy to MPH + ATX combination, with one-year follow-up.	2010 to 2014	7-17	12	Efficacy	None/none
Park (2024) [19]	Retrospective observational (chart review)	ADHD pediatric patients from one hospital in Korea, with at least two prescriptions of either MPH or ATX, or both combined after failing to improve on monotherapy.	December 2019 and February 2023	6-12	96	Efficacy	None/none

**Table 3:** Summary of findings for efficacy. ATX: atomoxetine; ADHD: attention-deficit/hyperactivity disorder; CGI-S: Clinical Global Impressions-Severity; MPH: methylphenidate; CMAP: combined ATX and MPH pharmacotherapy; COM: combination therapy.

First author (year)	Measurement criteria	Cohorts by intervention		Key outcome	Certainty of evidence	Comments
Clemow (2015) [15]	At least two time points within a three-year intake period: a minimum of 50 days between baseline and endpoint assessments for patients prescribed atomoxetine (ATX) as monotherapy or in combination with other ADHD medications.	Monotherapy (n = 77)	Combination therapy (n = 108)	No significant difference in mean Clinical Global Impressions-Severity (CGI-S) endpoint scores between the combination therapy (3.838) and monotherapy (3.849) cohorts after propensity score stratification matching.	⊕◯◯◯ Very low	Downgraded one level: considerable confounding effects to efficacy measurements from lack of non-ATX washout period in study design unlikely to be mitigated by propensity score stratification, some concern for selective outcome reporting, and some indirectness of evidence (i.e., ~96% of the combination therapy cohort had co-prescribed a stimulant with ATX, but ~24% also had ADHD drug permutations beyond ATX and one stimulant).
Ozbaran (2015) [18]	Two time points 12 months apart: before and after combined ATX and methylphenidate (MPH) pharmacotherapy (CMAP).	Before CMAP (n = 12)	After CMAP (n = 12)	Mean CGI-S score was significantly reduced (P = 0.003) from before CMAP (5.08) compared to after CMAP (3.08), with 75% of patients (9 out of 12) in the cohort showing a significant reduction in ADHD severity (i.e., CGI-S score reduction by at least 2).	⊕◯◯◯ Very low	Downgraded one level: from lack of control cohorts (i.e., ATX or MPH monotherapy) and small sample size.
Park (2024) [19]	Three time points within 39 months: “baseline” = medication-free state, “1st point” = after only monotherapy but before combined ATX + MPH therapy, “endpoint” = time of maximal improvement on combined ATX + MPH pharmacotherapy.	ATX Monotherapy (ATX) (n = 30)	Combination therapy (COM) (n = 34)	Within the COM cohort, there was no significant difference between the baseline CGI-S score (mean ± standard deviation) (5.18 ± 0.76), and the mean CGI-S score after only monotherapy (4.32 ± 0.81).	⊕⊕◯◯ Low	No net change in levels: adequate statistical methods and directness of evidence within the scope of a non-randomized study of interventions without conflicts of interest.
		MPH monotherapy (MPH) (n = 32)		However, there was a significant reduction (P < 0.001) in CGI-S score in the COM cohort after only monotherapy (4.32 ± 0.81) and after switching to combination therapy (2.32 ± 0.81).		
				There were no significant differences in endpoint CGI-S scores between all three cohorts (COM: 2.32 ± 0.81; MPH: 2.25 ± 0.80; ATX: 2.30 ± 0.70).		

**Table 4:** Summary of findings for adherence. MPR: medication possession ratio; ATX: atomoxetine; MPH: methylphenidate; COM: combined exposure; SEP: separate exposure; HR: hazard ratio; 95% CI = 95% confidence interval; CM: child monotherapy; CC: child combination therapy; AM: adult monotherapy; AC: adult combination therapy; ADHD: attention-deficit/hyperactivity disorder; A2A: alpha-2-agonist; LoT: length of therapy; OROS-MPH: osmotic-release oral system methylphenidate; OR: odds ratio.

First author (year)	Measurement criteria	Cohorts by intervention				Key outcome	Certainty of evidence	Comments
Bahn (2021) [14]	Adherence conditions: 10+ clinic visits or more than six months consecutively, with medication possession ratio (MPR) ≥ 0.8.	Atomoxetine (ATX) (n = 146)	Methylphenidate (MPH) (n = 627)	Separate exposure (SEP) (n = 106)	Combined exposure (COM) (n = 50)	The COM cohort had significantly longer treatment duration in years (mean ± standard deviation) (COM: 4.56 ± 3.72), relative to the monotherapy cohorts (ATX: 1.87 ± 2.28, MPH: 2.38 ± 2.78; P < 0.0001).	⊕⊕⊕◯ Medium	Upgraded one level: large effect size (HR < 0.5) in the absence of major risks of biases or uncontrolled confounders, per in-depth and overlapping statistical analyses with subgroup comparisons.
						The COM group showed a significantly lower hazard ratio (HR) (HR: 0.40, 95% CI: 0.27 to 0.58; P < 0.0001) for discontinuation relative to the reference ATX and MPH monotherapy group (HR: 1.00).		
Clemow (2015) [15]	Total prescription refill days over a three-year intake period, with refill dates beyond considered as “ongoing treatment at study completion.”	“Child monotherapy” (CM) (n = 51)	“Child combination therapy” (CC) (n = 50)	“Adult monotherapy” (AM) (n = 24)	“Adult combination therapy” (AC) (n = 43)	Mean “duration on treatment regimen" (in weeks) was longer in the combination therapy cohorts (CC: 44.0 ± 36.0, AC: 39.2 ± 29.6) than the ATX monotherapy cohorts (CM: 31.9 ± 27.6, AM: 33.3 ± 28.4), regardless of weight class (no statistical significance provided in study).	⊕◯◯◯ Very low	Downgraded one level: considerable confounding effects to adherence data from lack of non-ATX washout period in study design, considerable concern for selective outcome reporting, and some indirectness of evidence (i.e., ~96% of the combination therapy cohort had co-prescribed a stimulant with ATX, but ~24% also had ADHD drug permutations beyond ATX and one stimulant).
						Patients with “ongoing treatment at study completion” were proportionally higher in the combination therapy cohorts (CC: 86.0%, AC: 76.7%) than the ATX monotherapy cohorts (CM: 59.6%, AM: 66.7%), regardless of weight class (no statistical significance provided in study).		
Clemow (2016) [16]	All cumulative dates with filled prescriptions within the 365-day follow-up period count toward “length of therapy” (LoT), regardless of treatment gaps.	Monotherapy (n = 36,076)		Combination therapy (n = 1,548)		The mean "length of therapy" (LoT) in combination therapy patients (222.9 ± 99.9 days) was significantly longer (P < 0.0001) than in monotherapy patients (107.7 ± 101.4 days), regardless of ATX dosage or treatment naivety with non-ATX ADHD medications.	⊕⊕◯◯ Low	No net change in level: mitigated concerns for selective outcome reporting, some indirectness of evidence (i.e., unspecified ADHD drug permutations for combination therapy cohort during the study, beyond preindex assumptions of ~98% stimulants and ~11% A2As prescribed within the cohort).
Ishizuya (2021) [17]	Adherence conditions: prescriptions filled each month to maintain MPR ≥ 0.5 over 12 months.	Osmotic-release oral system methylphenidate (OROS-MPH) monotherapy (n = 1,139)		ATX addition within the first three months of OROS-MPH therapy (n = 214)		Based on the relative odds ratio (OR), patients with concomitant ATX prescription within the first three months of OROS-MPH therapy were significantly more likely to have poor adherence with OROS-MPH therapy (OR: 0.376, 95% CI: 0.273 to 0.517; P = 0.000), compared to patients on OROS-MPH without ATX as reference (OR: 1.00).	⊕⊕◯◯ Low	No net change in level: large effect size (OR < 0.5) counteracted by indirectness of evidence (i.e., unique legal and clinical factors against combination therapy in Japan, lack of adherence data beyond 12 months, or mean treatment durations for cohort comparison).

Efficacy: Study Methods and Findings

In the study by Clemow et al. (2015) [15], both adult and pediatric patients with their first ATX prescription, between the intake period of January 1, 2011, and December 31, 2013, were initially screened from two clinical sites in the US. Further inclusion criteria from the medical records review were as follows: patients must have been treated for at least 50 days with ATX, and patients must have completed efficacy assessments over at least two time points: once at the initial baseline date, and a second time after at least 50 days into ATX therapy, within the three-year intake period. The 191 patients that met all the above criteria, with ages approximately from six to 62 years, were then categorized into two main cohorts: monotherapy cohort (i.e., patients prescribed only ATX within the study period), or combination therapy cohort (i.e., patients prescribed ATX along with any other ADHD medications within the study period). Within the combination therapy cohort, it was stated that an amphetamine medication was co-prescribed to 62 (out of 109) patients (56.9%), while a methylphenidate medication was co-prescribed to 43 (out of 109) patients (39.4%). In combination with ATX, 76.1% of patients (83 out of 109) were co-prescribed one other ADHD medication, 21.1% (23 out of 109) were co-prescribed two other ADHD medications, and 2.8% (3 out of 109) were co-prescribed three other ADHD-indicated medications. Furthermore, a significantly higher proportion of patients (P < 0.0001) in the combination therapy cohort had prior treatment with ADHD-indicated medications (83.3%) compared to those in the monotherapy cohort (51.9%), and 75.0% of patients in the combination therapy cohort had used an ADHD-indicated medication, compared to 33.8% in the monotherapy cohort in the three months before treatment baseline assessment (P < 0.0001). To mitigate against these confounders and selection bias due to non-randomization, a propensity score stratification model via multivariate logistic regression was created to distribute patients into five different strata within each cohort, with patients in each stratum sharing similar characteristics, such as age (child = 6-12, adolescent = 13-17, adults = 18+), gender, clinical site, ADHD subtype, comorbidities, baseline CGI-S score, and treatment naivety. The six patients who did not fit properly into any propensity strata were excluded, resulting in 77 patients for the monotherapy cohort and 108 patients for the combination therapy cohort being accounted for in the propensity model. During baseline characteristic comparison prior to model redistribution, analysis of variance (ANOVA) was used for continuous variables, chi-square testing was used for categorical variables, and logistic regressions were used for dichotomous variables. Two variables were notable for significant differences between monotherapy vs. combination therapy: (a) baseline CGI-S scores (mean ± standard deviation (SD)) (3.9 ± 1.1 vs. 3.3 ± 1.2; P < 0.001), and (b) treatment-naive status (48.1% vs. 16.7%; P < 0.0001). Adjusted two-way analyses of covariance (ANCOVA) were then done post stratification to account for changes by the propensity model, producing the mean CGI-S scores for each cohort via the method of least squares (LS). Overall, both the ATX monotherapy and combination therapy cohorts were shown to have decreased ADHD severity at endpoint without significant differences when comparing the LS mean endpoint CGI-S scores between the monotherapy (3.849) and combination therapy (3.838) cohort regardless of age groups (LS mean difference: -0.011, 95% CI: -0.368 to 0.346; P = 0.9521). This was confirmed with endpoint CGI-S sub-scores between the monotherapy and combination therapy cohorts, which also showed no significant differences between child and adult ADHD questionnaire results among any of the four assessment axes (i.e., attention and organization, restlessness and control of activity level, emotional self-control, and well-being). However, within subgroup analyses, it was shown that ADHD patients with no comorbidities showed significant endpoint CGI-S score difference between combination therapy (3.729, n = 25) and monotherapy cohorts (4.552, n = 17) (LS mean difference: -0.823, 95% CI: -1.587 to -0.058; P = 0.0359). Another point of interest was the statement within the study that, prior to propensity model stratification, all 14 out of 14 patients in the “ATX monotherapy to combined therapy” group “continued to improve after switching,” but no other context, data, or statistics were provided.

In the study by Ozbaran et al. (2015) [18], the efficacy of ATX and MPH combined therapy was compared to prior monotherapies, with assessments from CGI-S scores by psychiatrists at the Ege University Child and Adolescent Psychiatry Department Disruptive Behavior Disorders Clinic in Turkey. Pediatric ADHD patients at the university clinic between 2010 and 2014 were followed throughout their ADHD outpatient treatments, with patients with “Mental Retardation” excluded by using the Wechsler Intelligence Scale for Children. Out of the 824 patients, a cohort of 12 patients aged seven to 17 years were selected for chart review and noted to have switched from either MPH or ATX monotherapy to combined MPH + ATX pharmacotherapy (CMAP). The 12-patient cohort had no comorbidities of bipolar disorder, autism spectrum disorder, or psychotic disorders, and were evaluated by psychiatrists once before switching from either ATX or MPH monotherapies to CMAP, and again after 12 months of CMAP during routine follow-up. Overall, there was a significant reduction (P = 0.003) in ADHD severity on average between the start of CMAP (mean CGI-S: 5.08, 5 = markedly ill) and one year after CMAP (mean CGI-S: 3.08, 3 = slightly ill). This was also supported by the CGI-Improvement (CGI-I) ratings alongside the CGI-S ratings, in which nine out of 12 patients in the CMAP cohort were given a CGI-I rating of 2 after one year at the endpoint, which corresponds with being “much improved” in ADHD treatment. The mean CGI-I score was 2.33 for the 12-patient cohort, which is between “2 = much improved” and “3 = slightly improved.” However, no standard deviations were reported for either mean CGI-I or CGI-S scores within this study.

In the study by Park et al. (2024) [19], ADHD patients aged six to 12 years with at least two prescription refills between December 2019 and February 2023 of the non-stimulant atomoxetine (ATX) and/or the stimulant methylphenidate (MPH), were selected from Keimyung University Dongsan Medical Center. Patients with autism, intellectual disability, epilepsy, or other severe medical and psychiatric diagnoses requiring hospitalizations were excluded from the study. Patients enrolled in the study were divided into three cohorts, each of which underwent further selection to form three groups of comparable sample sizes: stimulant monotherapy (MPH, n = 32), non-stimulant monotherapy (ATX, n = 30), and combination pharmacotherapy (COM, n = 34). The combination pharmacotherapy cohort and group consisted of former monotherapy patients of either ATX or MPH who had failed initial treatment and were offered new augmentation with MPH or ATX, respectively, as an aggressive treatment option. For categorical data such as the number of comorbidities, chi-square testing was used. With numerical data, either one-way analysis of variance (ANOVA) or the Kruskal-Wallis test was used, depending on normality in distribution. CGI-S scores were collected at “baseline” (medication-free state), “1st point” for the combination pharmacotherapy group (after monotherapy started but before switching to combined therapy), and “endpoint” (after completing monotherapy or combined therapy). To determine efficacy before and after therapy in each group internally, CGI-S scores were compared by either a paired sample t-test or Wilcoxon signed-rank test, depending on data normality. CGI-S scores were compared from “1st point” to “endpoint” with the combination pharmacotherapy group, while the scores were compared from “baseline” to “endpoint” with the monotherapy groups. To compare the degree of CGI-S score change between the three groups at their respective start points and endpoints, repeated-measures ANOVA was deemed suitable. Each group had their own demographic and clinical characteristics compared with the two other groups, which were considered without significant differences overall other than scores from the Korean version of the Child Behavior Checklist (K-CBCL) (mean ± SD) (COM: 67.62 ± 8.99, MPH: 64.85 ± 7.60, ATX: 63.52 ± 11.41; P = 0.027) and the prevalence of comorbidities (COM: 32%, MPH: 19%, ATX: 7%; P = 0.036). Among the results of this study, efficacy was demonstrated in the combination pharmacotherapy group after switching from monotherapy to combination therapy, as evident from the significantly decreased (P < 0.001) mean CGI-S score between the “1st point” (4.32 ± 0.81) and “endpoint” (2.32 ± 0.81). Also of note was that the mean “baseline” CGI-S score for all patients selected for the combination pharmacotherapy group was higher than that of the monotherapy groups (5.18 ± 0.76). The mean CGI-S score changes of the MPH group from “baseline” (4.63 ± 0.71) to “endpoint” (2.25 ± 0.80) were equally significant (P < 0.001) to that of the ATX group’s “baseline” (4.67 ± 0.84) to “endpoint” (2.30 ± 0.70). When compared with the combination pharmacotherapy group’s changes from “1st point” to “endpoint,” there were no significant differences in mean CGI-S score at the endpoint between all three groups (P = 0.142). As for predictive factors for combination therapy, subgroup analyses indicate significant attributions to high K-CBCL total scores (OR: 1.152, 95% CI: 1.020 to 1.300; P = 0.023) translating to “more prominent emotional and behavioral problems,” and positive comprehensive attention tests (CAT) for “poor visual attention” (OR: 5.816, 95% CI: 1.651 to 20.482; P = 0.006).

Adherence: Study Methods and Findings

In the study by Bahn & Seo (2021) [14], outpatients diagnosed with ADHD at the Kyung Hee University Hospital were initially screened for between January 2009 and December 2019, all of whom had two or more visits to the hospital clinic. Initially, 1,189 outpatients aged two to 74 years were included, but 260 were excluded from the analysis due to non-exposure to any ADHD medications despite receiving the diagnosis. Out of the remaining 929 outpatients who underwent ADHD treatment, four cohorts were created based on their treatment patterns: ATX monotherapy (ATX), MPH monotherapy (MPH), ATX + MPH therapies combined concurrently (COM), and ATX + MPH therapies at separate times (SEP). To be considered in adherence with therapy, patients must have either visited the outpatient clinic 10+ times or more than six consecutive months, while also having their MPR maintained at greater or equal to 0.8 cumulatively. MPR was calculated as the number of months patients were prescribed and refilled their medication, divided by the total number of months elapsed. Chi-square testing was used to identify differences in categorical variables between the treatment cohorts, such as age (<18 or ≥18), sex (male or female), and outcome (maintenance or discontinuation). To compare the mean treatment durations, the Kruskal-Wallis test was used across the four cohorts, with Bonferroni post hoc analyses to compare each cohort's pairings for differences. Simple Cox regressions, along with multiple Cox regressions that adjusted for age and sex, were conducted comparing the following: three treatment groups (ATX and MPH vs. COM vs. SEP), two age groups (<18 vs. ≥18), and two sex groups (male vs. female). Hazard ratios (HR) were reported for discontinuation vs. maintenance over their survival durations, with HR = 1.00 being assigned to the reference groups (i.e., ATX and MPH, male, age <18). Further multiple Cox regressions were also completed for treatment subgroups stratified by age and sex, with interaction analyses by the Wilcoxon rank sum test adjusted for age and sex to confirm them as either confounders or effect modifiers for adherence within each treatment group. During baseline population characteristic comparisons, there were no significant differences found in the age and sex distributions of the four treatment groups, but it was noted that the treatment duration (mean ± SD) in years for both the “COM” (4.56 ± 3.72, n = 50) and “SEP” (3.85 ± 2.98, n = 106) groups were statistically significantly longer (P < 0.0001 for both “COM” and “SEP”) compared to the ATX (1.87 ± 2.28, n = 146) and MPH (2.38 ± 2.78, n = 627) monotherapy groups. Based on multiple Cox regression adjusting for age and sex, it was notable that the “COM” group was 0.40 times as likely as the “ATX and MPH” group to discontinue treatment (HR: 0.40, 95% CI: 0.27 to 0.58; P < 0.0001) for the “COM” group, relative to the reference HR of 1.00 for the “ATX and MPH” group. From the subgroup multiple Cox regressions comparisons, there was a particularly large difference among the COM group’s female patients (HR: 0.19, 95% CI: 0.05 to 0.70; P = 0.0131) relative to males in further decreased risk of treatment discontinuation, which was further confirmed via interaction analysis between female to male COM group patients (HR: 0.34, 95% CI: 0.12 to 0.99; P = 0.0481).

In the study by Clemow et al. (2015) [15], the overall propensity model patients were first divided into two age/weight classes and then by therapy groups for treatment pattern analysis. Four subcohorts were thus created: “children and adolescents weighing ≤154 lb, in monotherapy” (CM, n = 51), “children and adolescents weighing ≤154 lb, in combination therapy” (CC, n = 50), “children and adolescents weighing >154 lb and all adults, in monotherapy” (AM, n = 24), and “children and adolescents weighing >154 lb and all adults, in combination therapy” (AC, n = 43). The mean "duration on treatment regimen" in weeks (mean ± SD) was as follows: CC: 44.0 ± 36.0; AC: 39.2 ± 29.6; CM: 31.9 ± 27.6; AM: 33.3 ± 28.4. Another adherence-related measure was the percentage of patients with “ongoing treatment at study completion” who maintained prescriptions even beyond their study end date. The percentages for “ongoing treatment at study completion” for combination therapy were as follows: 86% (43 out of 50) for “children and adolescents weighing ≤154 lb,” and 76.7% (33 out of 43) for “children and adolescents weighing >154 lb and all adults.” The percentages for “ongoing treatment at study completion” for ATX monotherapy patients were as follows: 59.6% (31 out of 51) for “children and adolescents weighing ≤154 lb,” and 66.7% (16 out of 24) for “children and adolescents weighing >154 lb and all adults.” Thus, the combination therapy cohort (n = 93) had a longer mean duration on treatment regimen and higher proportions of patients in maintenance at the end of the study than the ATX monotherapy cohort (n = 75), regardless of age/weight class difference. However, no statistical comparisons were done within the study to determine significance for either data set of adherence metrics.

In another study by Clemow et al. (2016) [16], 37,624 adult ADHD patients having at least one pharmacy claim for atomoxetine were screened from January 1, 2006, to September 30, 2013, within the Truven Health Marketscan Commercial Claims Database for the US. The de-identified prescription data of each patient was required to show them to be naïve to ATX treatment six months before the date of their first ATX prescription, with the first ATX prescription date within the study period defined as the index date. All patients must also have been covered with medical and drug benefits from six months before and 12 months after the index date to be eligible for study inclusion. Any patients found to have >30 days of continuous overlap with another ADHD medication prescription (i.e., amphetamine, methylphenidate, or alpha-2 adrenergic agonists) within the study period were classified under the combination therapy cohort. The baseline characteristic “Preindex ADHD Medication Use” data revealed the ADHD medications prescribed alongside ATX that were tracked, consisting of mainly stimulants of varying timed-release formulations (i.e., short-acting, long-acting, prodrug, etc.). The ATX monotherapy cohort and the combination therapy cohort were then each subdivided into those who had reached the recommended adult ATX dose of 80 mg/day or those who did not reach the ATX dose of 80 mg/day. Without the assumption of normal distribution within certain data sets, the Wilcoxon rank-sum test was used for comparing the sub-cohorts of two different ATX dosages, such as for mean length of therapy (LoT) and other measures of dosing patterns. LoT was defined in the study as “all prescription claim fill days over the 365-day follow-up period,” regardless of use frequency or gaps. From the data relevant to adherence in the study, it was noted that the combination therapy cohort had longer mean LoT than the ATX monotherapy cohort, regardless of ATX dosage. The mean LoT was 222.9 ± 99.9 days (mean ± SD) for the 1,548 combination patients, which was significantly greater than the mean LoT of 107.7 ± 101.4 days for the 36,076 monotherapy patients (P < 0.0001). In subgroup analysis, an observation was made that being unexposed to any ADHD treatment at least six months prior to ATX monotherapy (defined as being “Treatment Naïve”) significantly increased patient mean LoT, compared to those who had non-ATX ADHD treatment within six months prior to ATX monotherapy (defined as being “Not Treatment Naïve”). The mean LoT was 112.0 ± 103.0 days for the 25,960 “Treatment Naïve” patients, which was significantly greater than the mean LoT of 96.7 ± 96.3 days for the 10,116 “Not Treatment Naïve” patients (P < 0.0001). Statistical significance was not observed, however, when comparing “Treatment Naïve” with “Not Treatment Naïve” patients within the combination therapy cohort.

In the study by Ishizuya et al. (2021) [17], pediatric ADHD patients in Japan with private insurance prescription records nationwide were compiled between December 2007 and May 2015. Only those who were not prescribed any form of methylphenidate drugs within one year prior to their first osmotic-release oral system methylphenidate hydrochloride (OROS-MPH) prescription, and only those with observational data every month for at least one year were included in the study. A total of 1,353 children aged six to 17 years met all criteria for inclusion, where patient adherence was represented in the criteria of MPR being ≥ 0.5. MPR was calculated as the number of months with filled OROS-MPH prescriptions, divided by the number of months in therapy before the follow-up at the endpoint of the study (i.e., 12 months). A two-tailed multivariable logistic regression analysis was also performed, based on different patient characteristics identified as risk factors that may potentially contribute to poor adherence to OROS-MPH therapy. Out of the 1,353 patients, 214 patients had the concomitant use of atomoxetine in the first three months of OROS-MPH therapy, while the remaining 1,139 patients served as the reference OROS-MPH cohort unexposed to ATX. In the concomitant ATX usage cohort, 36% (77 out of 214) patients were deemed to be adherent (MPR ≥ 0.5) to their OROS-MPH therapy, while 64% (134 out of 214) patients were below the MPR of 0.5 to be considered adherent. In the reference OROS-MPH cohort unexposed to ATX, 60.1% (685 out of 1,139) patients were deemed to be adherent (MPR ≥ 0.5) to their OROS-MPH therapy, while 39.9% (454 out of 1,139) patients were below the MPR of 0.5. The MPR of the concomitant ATX + OROS-MPH therapy cohort was 0.38 ± 0.34 (mean ± SD), which is less than that of the reference OROS-MPH cohort (0.54 ± 0.32) unexposed to ATX. Among the risk factors in the multivariable logistic regression of the adherence data, there exists an odds ratio (OR) of 0.3760 compared to the adherence of the reference OROS-MPH therapy group to ATX concurrent using subgroup. The 95% CI for the OR was between 0.273 and 0.517, while the P-value was reported as “P = 0.0000.” Overall, the odds ratio indicates concomitant ATX prescription with OROS-MPH is a statistically significant risk factor for poor adherence with OROS-MPH therapy in general.

Discussion

Efficacy

Clemow et al. (2015) [15] used the most advanced multivariate statistical methods out of all three studies, with propensity score matching for patients across two clinical sites, in an effort to reduce selection bias and other confounders. However, the fact that no washout period was required for the eligible sample population introduces considerable confounders, as shown by the baseline CGI-S score differences, which may not be compensated by any statistical adjustments. Beyond the study design itself, there were inherent concerns with selective outcome reporting as well, such as the declaration of 14 patients that “continued to improve after switching” from ATX monotherapy to combined therapy with regard to ADHD severity in the discussion section. Coincidentally, the study’s patient distribution diagram showed 14 patients under the sub-cohort of “monotherapy to combination therapy group,” alongside the sub-cohort of 95 patients in the “combination therapy group”; both of which fall under the “combination therapy” cohort for aggregated analysis. This might suggest that all 14 out of 14 patients in the “monotherapy to combination therapy group” could have improved even more beyond the end score of each patient’s study periods, and yet there did not exist any further elaborations with data, or investigation with statistical analyses. This is contrasted with the level of detailed reporting and analyses for evidence to prove the lack of efficacy in combination therapy vs. ATX monotherapy, thus increasing the risk of reporting bias in line with the declared conflict of interest in the study and author funding. Finally, another factor to consider was the indirectness of evidence due to the “combination therapy” cohort consisting of aggregate data of different ADHD medication permutations, as stated: “In addition to ATX, 76.1% of patients were prescribed one other ADHD medication, 21.1% were prescribed two other ADHD medications and 2.8% were prescribed three other ADHD-indicated medications.” Despite the majority of patients (105 out of 109, 96.3%) in the combination therapy cohort being prescribed stimulants, the impact of 23.9% of patients with unspecified drug combinations beyond ATX and a stimulant as a confounder was uncertain. Overall, the certainty of evidence was considered “Very Low” regarding this study’s efficacy results, due to all the above-mentioned points.

Ozbaran et al. (2015) [18] was an industry-independent study, which showed statistically significant improvement in efficacy for the 12-patient cohort that received combined ATX + MPH therapy compared to monotherapies, after a follow-up period of one year. The level of detail available for each of the 12 individual patients, including age, gender, diagnosis, sequence, and dosages going from monotherapy to combination therapy, along with side effects, was unsurpassed by any other study. However, beyond being a study of such a small sample size from a single clinical site, the statistical methods used were also somewhat vague. The only statistical comparisons made were between before (monotherapy) and after (combined ATX + MPH therapy) within the same cohort, without any identifiable control groups remaining on either monotherapy. Overall, the certainty of evidence in this study was considered “Very Low” in its strength to answer this review’s research question.

Park et al. (2024) [19] was an industry-independent study that was able to use fairly robust and transparent statistical methods to demonstrate that for treatment-resistant patients on either MPH or ATX monotherapy, combined pharmacotherapy could achieve the same significance of reduction in ADHD severity compared to their monotherapy counterparts when prior monotherapies have failed for any number of reasons. The transparency in the sequence of switching from monotherapy to combined therapy and the transparency in the timing of the efficacy measurements, along with the use of repeated measures ANOVA, were unique in their robustness to ensure that the pediatric patient data had internal validity. However, the study had limitations in being a single-site study, with the study design admittedly ceasing to capture further changes in CGI-S scores beyond the point of maximal improvement. Even though this study had the strongest evidence for efficacy out of the three, the certainty of the evidence was still considered “Low.”

After considering the certainty of evidence across all three studies, it seemed likely that while the general ADHD population did not find significantly greater benefit in combination therapy of ATX and stimulants like MPH compared to either monotherapy, there existed a small subpopulation of ADHD patients that benefited from combined ATX and MPH therapy, when prior monotherapy had proven insufficient. And yet, the current overall quality of evidence leaned toward being “Very Low.”

Adherence

Bahn & Seo (2021) [14] demonstrated the most transparent and rigorous use of statistical testing to investigate adherence differences between monotherapy vs. combination therapy, with the separation of categories within combination therapy by concurrent (COM) vs. non-concurrent (SEP) use of ATX and MPH, across the longest study period of 10 years. The authors and the study also had no perceivable industrial conflicts of interest. The combination of single vs. multiple Cox regressions, and the confirmation of subgroup multiple Cox regression results with Wilcoxon rank-sum tests as part of interaction analyses in age and sex shows the level of due diligence that the authors showed in controlling for confounders. The authors even went as far as to confirm that there were no significant differences between the 929 included and 260 excluded non-treatment patients from analysis based on age and sex. Thus, the reportedly large effect size observed with the COM group in reducing discontinuation of therapy relative to the ATX and MPH monotherapies could be seen as robust enough to be upgraded in quality by one level. The biggest limitation of the study was in its sample population being from only one hospital in Korea, which weakens the generalizability of the results, but the certainty of evidence could still be considered “Medium” when taken into context.

Beyond the assessment already made by Clemow et al. (2015) [15] regarding study design limitations and indirectness of evidence, there were even greater concerns with selective outcome reporting in adherence results. Across the two cohorts, there were 101 patients in the “children and adolescents weighing >154 lb and all adults” category, and 67 patients in the “children and adolescents weighing ≤154 lb” category. However, assuming that there were 185 patients total in the propensity model population, with 168 patients divided into the two age/mass categories, no details were provided on the 17 patients that were missing from both categorizations. Even still, the combination therapy cohort had a longer mean “duration (weeks) on treatment regimen,” and greater “% ongoing treatment at study completion” compared to the monotherapy cohort, regardless of age/mass class difference, but no further analyses were provided beyond the weeks and percentages shown on the table. However, in this review’s efforts for inferential statistics, the chi-square test with Yates correction (chi-square statistic: 6.9592) revealed a significantly greater (P = 0.0083) proportion of patients with “ongoing treatment at study completion” in the combination therapy cohort compared to the monotherapy cohort (OR: 3.96, 1.4922 to 10.5259; P = 0.0057) within the “child” age/weight class, with borderline large effect size (i.e., if OR > 4). Although it was understood that the main focus of this study was on efficacy rather than adherence, there were methodological inconsistencies that were not clearly explained. Overall, the certainty of evidence with this study’s adherence results was considered “Very Low” due to all the above-mentioned points.

With Clemow et al. (2016) [16], patient adherence data appeared to show that the combination therapy cohort of 1,548 patients had statistically significantly longer mean length of therapy than the monotherapy cohort of 36,076 patients, regardless of ATX dosage or treatment naivety status to non-ATX ADHD medications. Compared to Clemow et al. (2015) [15], this study appeared better designed to control for treatment naivety and appeared to be more balanced in reporting relevant results both favorable and unfavorable despite the unchanged conflict of interest. Furthermore, there is strength in the sample size and geographic coverage of this study due to its use of a national prescription database for private health insurance. However, there are also drawbacks to the data being less generalizable due to socioeconomic bias, and lacking ability to capture actual oral intake of medications by patients. Moreover, there are uncertainties due to a lack of prescription data during the study for the combination therapy cohort after the index period began. Yet, based on assumptions from the “Preindex ADHD Medication Use” breakdown under demographic and clinical characteristics, the vast majority of patients in both the monotherapy cohort (36,855 out of 37,624, 98.4%) and the combination therapy cohort (1,523 out of 1,548, 98.4%) were treated with at least one stimulant regardless of formulation, with a2-agonists use appearing to be a relatively mild confounder (monotherapy cohort: 600 out of 37,624, 1.7%; combination therapy cohort: 169 out of 1,548, 10.9%). Overall, there was no compelling or outstanding imbalance of reasons to change the certainty of evidence rating from the default of “Low” for this study.

Ishizuya et al. (2021) [17] gathered 12 months of prescription data for each of the 1,353 insured pediatric ADHD patients, with 1,139 patients who were prescribed OROS-MPH without ATX and 214 patients who were also prescribed ATX within the first three months of OROS-MPH therapy. The analysis of patient adherence shows that concomitant ATX exposure is associated with significantly lowered MPR on OROS-MPH therapy. Based on the reported odds ratio, the patients' concomitant use of ATX in the first three months of OROS-MPH therapy is 2.66 times more likely to have poor adherence with OROS-MPH compared to those who did not use ATX concurrently. However, despite the large effect size, the study did not further explore how the concomitant ATX prescription led to poor adherence to MPH, which the authors admit could be due to merely dose-reduction in the augmentation process. The strength of this study lies in its use of a multivariate logistic regression model with two-tailed analyses, done upon a sample population size that was the second largest out of all studies included in this review. However, this is mitigated by the lack of data beyond MPR across the 12-month period and the lack of comparison with other treatment groups, such as actual mean treatment durations for combined ATX and OROS-MPH vs. OROS-MPH monotherapy vs. ATX monotherapy. Yet, even the inclusion of such additional metrics might not produce meaningful results, given the major limitation mentioned by the authors in Japanese clinical guidelines, which considers both OROS-MPH and ATX as first-line treatment options. Furthermore, the authors mentioned the strictness of Japanese laws regarding narcotics, such that a circulation management committee exists to limit the number of qualified prescribers and total prescription of controlled stimulants. Therefore, with ATX and stimulants both considered first-line treatments and with stimulants facing such prescription barriers in Japan, the incentive for co-prescribing ATX and a stimulant was further reduced compared to completely switching from stimulant to ATX. Finally, as a review of prescription records instead of patient charts, factors such as patient comorbidities, adverse events, and actual oral intake as prescribed were not captured. Overall, the number of study limitations was balanced against any observable effect size, and thus the certainty of evidence remains unchanged at “Low.”

After considering the certainty of evidence across all four studies, it was concluded that there was more evidence toward increased adherence with combination therapy compared to either ATX or MPH monotherapy alone, although the overall certainty leaned toward “Low.” Bahn & Seo (2021) [14] provided the most direct comparison of combination therapy to both monotherapies without conflicts of interest, with the study’s transparent and rigorous statistical methods, moderately large sample size, and long study period. Both studies by Clemow et al. (2015, 2016) [15,16] indicated that ATX combination with stimulants had a longer mean adherence period than ATX monotherapy alone overall despite conflicts of interest in funding, with the 2016 study being more robust in controlling for confounders out of the two, and had the largest sample population size out of all studies as well. The only counterexample for adherence was Ishizuya et al. (2021) [17], which focused mainly on OROS-MPH and concluded that concomitant prescription of ATX in the first three months was a significant risk factor for poor OROS-MPH adherence with a large effect size. However, the lack of comparison data for other treatment cohorts at longer time periods, along with Japan’s unique legal and clinical barriers to combined ATX and MPH therapy, rendered the certainty of evidence in this study as “Low” in its applicability to ADHD patient populations elsewhere.

Limitations

It must be noted that there are limitations to the interpretation of this review, both from its methods and eligibility criteria. Although considered lower quality evidence by design, the exclusion of case series and non-human studies might contribute to publication bias. Among the six studies that met eligibility criteria to be relevant to the research question, all were retrospective cohort studies and none were randomized controlled trials (RCTs). Besides the default risk of bias inherent in the non-randomized nature of the included studies, two of the six had considerable conflicts of interest due to industrial funding to both the authors and the study themselves. Across multiple studies, the heterogeneity of sample population characteristics (e.g., population sample size, age range, geography, etc.), adherence metrics (e.g., different MPR thresholds, different time periods, etc.), and interventional biases (i.e., ATX vs. OROS-MPH as the main focus of certain studies) were all contributors to the indirectness of evidence in applicability. With most reported summary statistics being based on non-parametric tests among the studies, the lack of raw data availability for each study precludes most new inferential statistics about their individual effect sizes from being reliable, thus also making further meta-analyses unfeasible for this review overall.

In terms of efficacy, the difference in study designs (e.g., washout periods (if any), treatment naivety conditions, etc.), along with the varying lengths in assessment periods between studies, also prevented the generalizability of the results, even for identified ADHD subpopulations with common predictive factors. The lack of detailed dosage-stratified data in all but one study, which only focused on ATX as per its industry funding interests, also left the time-dose response as a largely unexplored factor in this review.

In terms of adherence, beyond the difference in criteria of treatment gaps, there were non-quantifiable factors to be aware of as well. Socioeconomic factors such as differences in healthcare coverage and insurance policies, along with regulatory and treatment guideline differences between nations regarding controlled substances, were all possible confounders that could affect the outcomes even with seemingly standardized definitions and shared metrics. Another factor that affected adherence not explicitly explored in this review was tolerability and adverse events, which might offer further qualitative insights that were considered beyond the quantitative scope of this review.

## Conclusions

The overall argument for the effectiveness of combined ATX and stimulant therapy remains unchanged from the last literature review in 2013: some but not all patients who have tried but failed either monotherapies may benefit from the ATX and stimulant combination. And yet, for those patients who do find benefit, there is some argument that increased adherence is among them. Past reviews have indicated that atomoxetine supplementation of stimulants may be beneficial for those with ADHD and comorbidities of anxiety, sleep, or tic disorders. However, these recommendations are largely based on historical broad prescription patterns and retrospective clinical observations, rather than any dedicated prospective quantitative studies.

For future reviews of the same research question to be more meaningful, RCTs specific to ATX combinations with each individual stimulant formulation must be conducted separately, with the research question narrowed to focus on patients with different characteristics (e.g., ADHD subtypes, comorbidities, etc.) as historically indicated by past literature. In protocol design for combination therapies, the monitoring of two medication dosage variables over time, along with accounting for varying patient responses to treatment, are all challenges to overcome. The collaborations of public hospitals around the world dedicated to a multi-site RCT, with minimal funding and influence from private industry interests, would be the ideal scenario. Otherwise, the meta-analyses of many smaller RCTs following the same standardized protocol may be the relatively more practical option. Regardless of study methodology, advancements in neuroimaging techniques and biomarker identification may allow better future study design, one able to identify and follow subsets of ADHD patients over extended follow-up periods. With more specific and powerful conclusions made in a controlled setting, these can then be compared with real-world cohorts for more generalizable clinical correlations in improving long-term outcomes.
